# The functional landscape of the appendix microbiome under conditions of health and disease

**DOI:** 10.1186/s13099-025-00696-2

**Published:** 2025-06-01

**Authors:** Md Shahjalal Sagor, Tarequl Islam, Noshin Tabassum Tamanna, Md. Kamrul Islam Bappy, Danishuddin, Md Azizul Haque, Maximilian Lackner

**Affiliations:** 1 Department of Microbiology, Jagannath University, Dhaka, 1100 Bangladesh; 2 Department of Microbiology, Noakhali Science and Technology University, Noakhali, 3814 Bangladesh; 3 Department of Pharmacy, Noakhali Science and Technology University, Noakhali, 3814 Bangladesh; 4 Department of Biotechnology, Yeungnam University, Gyeongbuk, 38541 Republic of Korea; 5 Department of Industrial Engineering, University of Applied Sciences Technikum Wien, Hoechstaedtplatz 6, 1200 Vienna, Austria

**Keywords:** Appendix microbiome, Gut health, Appendectomy, Neurological disorders, Immune system, Therapeutic potential

## Abstract

Traditionally regarded as a vestigial organ, the appendix is now being reevaluated for its significant function in health and nutrition of humans. Serving as a “safe house” for beneficial, desired gut bacteria, the appendix is protected by resilient biofilms that create a secure environment. This makes the appendix a”basin” for gut microbiota (GM), replenishing the microbial population following disruptions from infections, antibiotic use, or inflammatory bowel disease (IBD). Beyond simply hosting bacteria, the appendix has an active role in functions of the immune system. Disruption of the Appendix Microbiome (AM), such as through appendectomy, was found to result in lowered diversity of gut microorganisms and an increased risk of various diseases. The potential therapeutic applications of the AM are a particularly promising area of research. The appendix’s unique microbial environment and its impact on immunity open new avenues for treatments. These include modulating GM to improve cancer treatment outcomes, mitigating IBD, regulating metabolic pathways in obesity and diabetes, influencing neurotransmitter production in neurological disorders, and addressing cardiovascular and autoimmune diseases. This review highlights the appendix’s transformation from a misunderstood organ to a critical component of gut health and immunity. It explores the function of the human appendix as a resilient reservoir for desired microorganisms, and its role in disease progression. Furthermore, it examines the potential therapeutic applications of AM, presenting exciting opportunities for future research and treatment innovations.

## Statement of significance

The Appendix and its Microbiome (AM) that were previously thought of as a useless part of the human body have a deep relation with health and diseases. In this review articles, we broadly discuss factors affecting the function and composition of AM, diseases associated with AM dysbiosis, and its therapeutic potential. Since few studies have addressed the health impacts of the AM, this review offers a comprehensive overview, covering nearly all aspects of the relationship between the GM and AM, and provides noteworthy insights into their roles in health and diseases.

## Introduction

The human appendix, once thought to be a vestigial organ, has newly attracted interest for its possible function in maintaining healthy gut microbiota [1, 2]. The term "appendix" comes from the Latin word "appendere," which means "to hang upon" [3]. Historically, the appendix was named because it hangs on to the cecum, which resembles a little appendage suspended from the large structure. This etymology reflects the appendix's presumed function as a secondary addition to the gastrointestinal tract [4, 5]. Research suggests that the appendix functions as “sanctuary” for beneficial gut bacteria, protecting them and aiding in their replenishment after disruptions, such as diarrheal illness [6]. This bacterial reservoir may be crucial for restoring gut flora, which is essential for various bodily functions, including immune response and digestion [6, 7].

The appendix has evolved in an independent way across different species, including rodents, primates,, and marsupials [8–11]. The recurrence of evolution suggests that the appendix has important functions, rather than being a vestigial organ [9]. Research recommends that the appendix has followed an independent evolution in various mammals, which is a clue for its functional role [9, 12]. It appears that the appendix has been present as an element of mammals’ digestive system for a very long time of at least 80 million years [13, 14]. This is a clue for a selective advantage by this organ [12].

In a healthy state, the AM helps keeping a balanced gut flora, which is essential for the overall body function [15]. In the case of a disease, alterations in AM composition can contribute to or exacerbate conditions like acute appendicitis, which is linked to lowered microbial diversity and a surge in pathogenic bacteria [6, 16]. Furthermore, appendix removal (appendectomy) has been linked to changes in the GM and is believed to cause long-term impacts on human health, including an elevated risk for cancer and certain neurological disorders [7, 17–20].

The gut-brain axis (GBA) connects the enteric and central nervous systems, involving hormonal, immunological, and neuronal pathways that together regulate gastrointestinal (GI) function, mood, and cognitive processes [21, 22]. It can be considered a bidirectional network of communication. A crucial component of this axis is the microbiome, consisting of trillions of microorganisms in the gastrointestinal (GI) tract that have a profound impact on the host's health and disease states [23]. Emerging studies suggest that the AM may influence the GBA. The GM can directly impact brain function and behavior, potentially affecting neurodevelopmental and psychiatric disorders [24, 25]. Specific microbial species in the AM are involved in neurotransmitter production. For instance, *Lactobacillus* and *Bifidobacterium* species produce GABA (gamma-aminobutyric acid), which helps regulate anxiety and stress responses [26]. *E. coli* produces serotonin, an important molecule [27]. *Enterococcus faecalis* produces dopamine, essential for reward and motivation pathways in the brain [27]. Clostridium species set free SCFA (short-chain fatty acids) like butyrate. These exhibitanti-inflammatory effects and can influence brain health [28]. As a microbial reservoir, the appendix may serve a vital function in regulating the GBA, particularly during periods of gut microbial imbalance [29]. The production of neurotransmitters by specific microbial species highlights the complex relationship between the gut and brain, with imbalances potentially resulting in neurological disorders. For instance, reduced GABA and serotonin levels are associated with depression and anxiety disorders [26], while alterations in dopamine production are linked to Parkinson's disease [27]. Furthermore, changes in SCFA production due to AM dysbiosis can impact cognitive function and contribute to Alzheimer's disease [28]. Santos et al. have found that stress is a potentiator of symptom severity in these disorders via the brain-gut-microbiota axis. This highlights the significant clinical implications of understanding the interaction between chronic psychological stress, gut microbiota, and the GBA in mental health and digestive diseases [30]. These insights underscore the significance of the AM in maintaining gut-brain communication and highlight its potential therapeutic implications for neurological health.

The precise mechanisms through which the AM interacts with various bodily functions are still being explored. However, it is hypothesized that the appendix may contribute to health by modulating the immune system, as it contains lymphoid tissue that supports the growth of beneficial gut bacteria. It is also likely that the appendix influences health by acting as a reservoir for bacteria that produce neurotransmitters or their precursors, potentially influencing mood and cognitive functions [22, 23, 31].

The AM is gaining recognition to impact health and diseases. Its potential influence on health offers new insights into conditions ranging from GI disorders and cancers to mental health issues. As research continues to resolve the complexities of the microbiome and its relations with the human body, the appendix may no longer be seen as a redundant organ but as a vital contributor to overall well-being.

In this review, the authors discuss factors affecting AM function and composition, diseases associated with AM dysbiosis (a microbial imbalance in the appendix or gut microbiome), and its therapeutic prospects. Throughout the review, the term “GM” was used frequently, as the GM and AM share similarities at the phylum and genus levels, although their composition differs. Since few studies have addressed the health impacts of the AM, this review offers a comprehensive overview, covering nearly all aspects of the relationship between the GM and AM, and it thereby provides valuable insights into their roles in health and diseases.

## Methodology

**Fig. 1:**
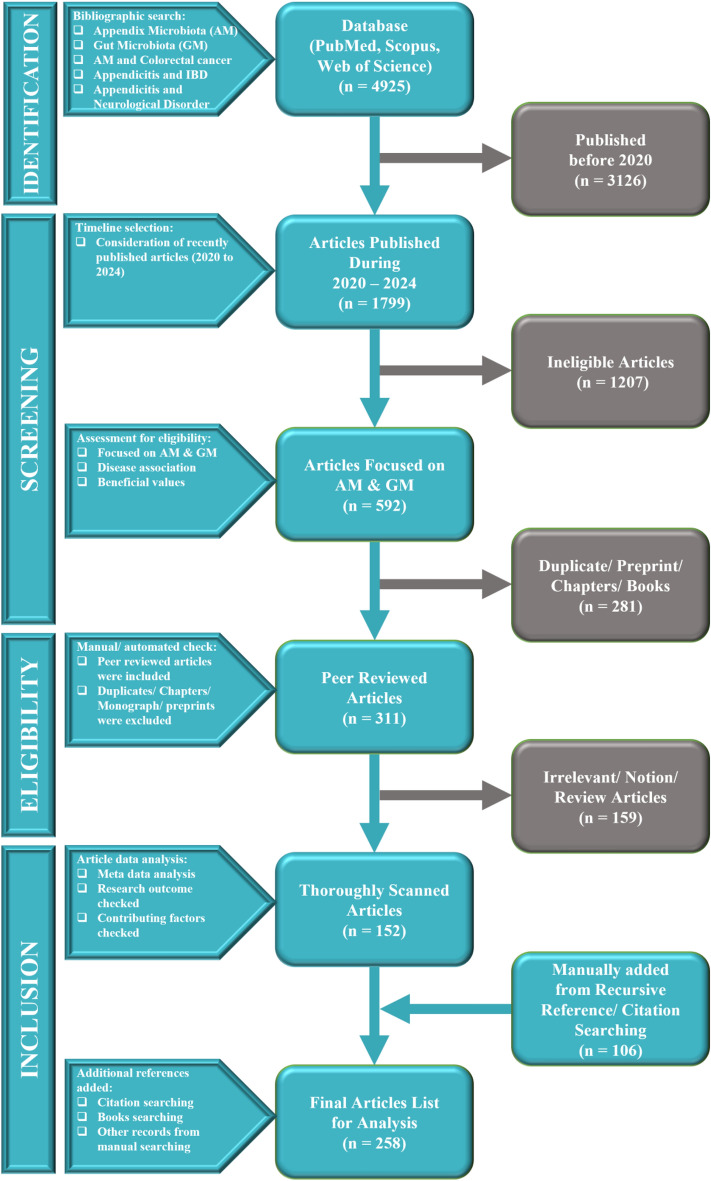
Article selection process for review. The flowchart of the approach for selection of research articles was adapted from the PRISMA flow chart by Moher et al. The chart illustrates the systematic identification, screening, and inclusion of articles to be analyzed. It encompasses stages like identification from bibliographic databases, screening based on publication timeline and relevance, eligibility checks through peer reviews, and final inclusion for thorough analysis. The number of articles retrieved, included, and excluded at different stages is stated in parentheses

This review thoroughly examines the determinants of AM function and composition, AM dysbiosis disease, and its therapy. An extensive literature search and meta-analysis was conducted with well-established databases and tools such as Web of Science, PubMed, Scopus, and Dimensions AI tools (Fig. 1) [32, 33]. The search was filtered with 2020 to 2024 published studies that examined the appendix, gut microbiome (GM), and AM functions in health. Some specific keywords like “Appendix microbiota”, “Gut microbiota”, “Appendix microbiota and colorectal cancer”, “Appendicitis and inflammatory bowel disease”, and “Appendicitis and neurological disorder” were used to search relevant articles.

To ensure the quality and relevance of included studies, specific exclusion and inclusion criteria were also used. The articles were screened based on their research strengths, peer-review status, and relevance to our review focus. Specifically, studies that discussed the function of the appendix in gut immunity and health, explored the impact of the appendix on disease progression, or investigated therapeutic applications of the AM were given importance. Articles that published between the years 2020–2024 received priority to capture the recent trends in the field. Non-peer reviewed studies, non-AM or GM specific articles, published before 2020, and duplicates/preprints were excluded from analysis.

Data extraction involved meticulous review of the abstracts and texts of the included articles to ensure their relevance and quality. Published data were gathered on different themes, including the appendix as a reservoir for defensive bacteria, impact of appendix removal (appendectomy) on diversity of gut microbiota, immunological functions of the appendix, and therapeutic application of the AM in various diseases. The influence of the appendix on disease progression was also a focal point of the analysis.

**Fig. 2:**
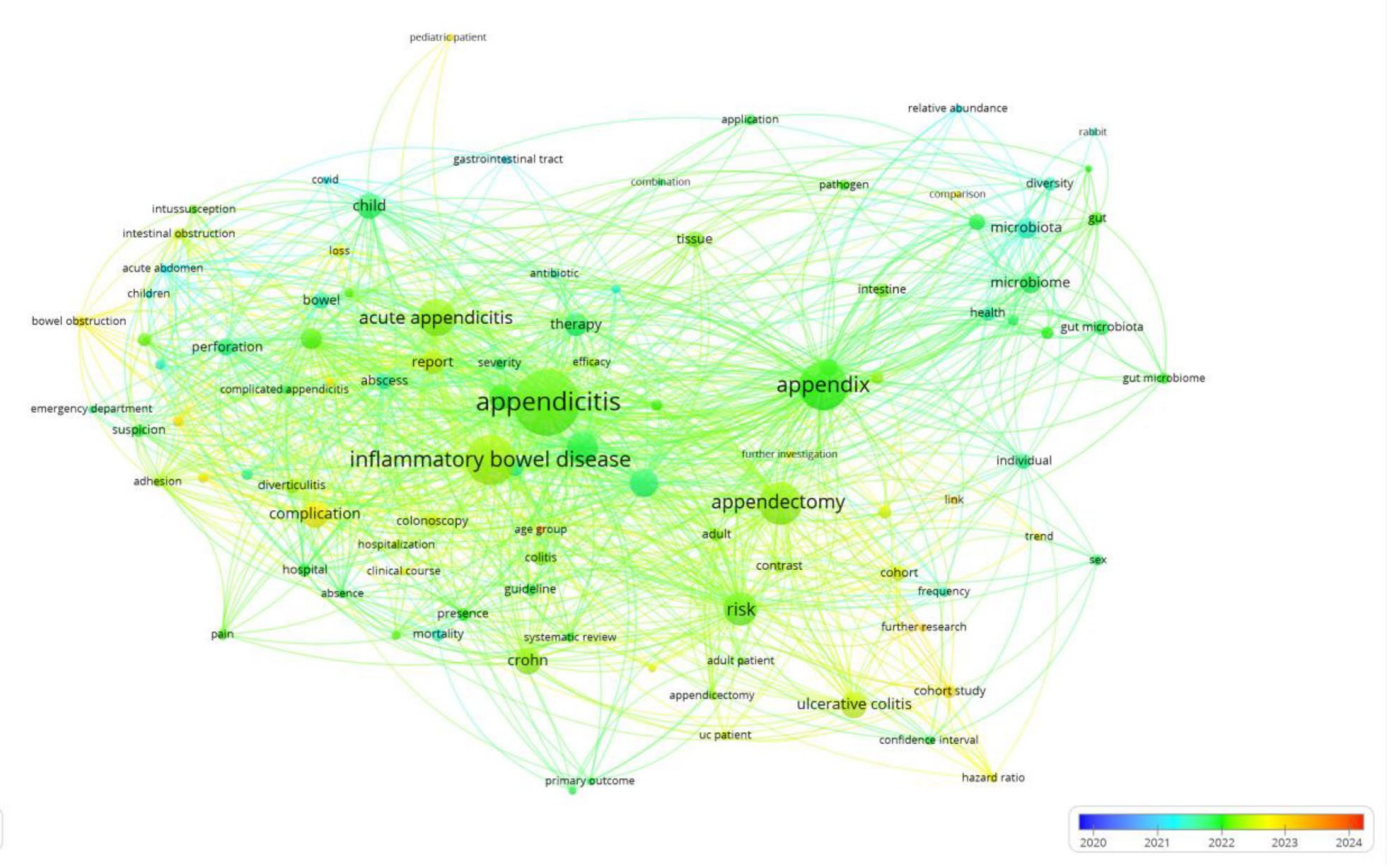
Bibliometric analysis of AM literature and related diseases research. VOSviewer version 1.6.20 & Dimensions research publication database were employed for bibliographic network analysis, mapping the relationship of key terms in AM research from 2020 to 2024. The analysis includes 152 studies and 100 keywords. The blue-to-red gradient represents the timeline of 2020 to 2024, describing the trend in research interest in recent time. The figure highlights the relationship between key terms in AM research. Central terms include “appendicitis”, “appendectomy”, “IBD”, and their complications

To judge the effect of AM on public health, a bibliographic analysis of recent research was conducted using VOSviewer (Fig. 2). The analysis was conducted with VOSviewer version 1.6.20 and Dimensions research publication database with 152 published articles in the period from 2020 to 2024 on AM and related diseases [32, 34]. The analysis was done with 100 keywords extracted from the publications and yielded four clusters in key words.. The network shows the geographical and time spread of the research, with teamwork in addressing AM and its impact..The findings of the network analysis and literature review were combined to give an overview of the appendix's role in gut immunity and health. The analysis brought out the transformation of the appendix from a mysterious organ to an essential component of gut health. It addressed the appendix as a healthy reservoir that is strong, the impact of disruption of the appendix on disease occurrence, and the therapeutic value of the AM in optimizing health outcomes.

## Factors affecting appendix and/or gut microbiome

**Fig. 3:**
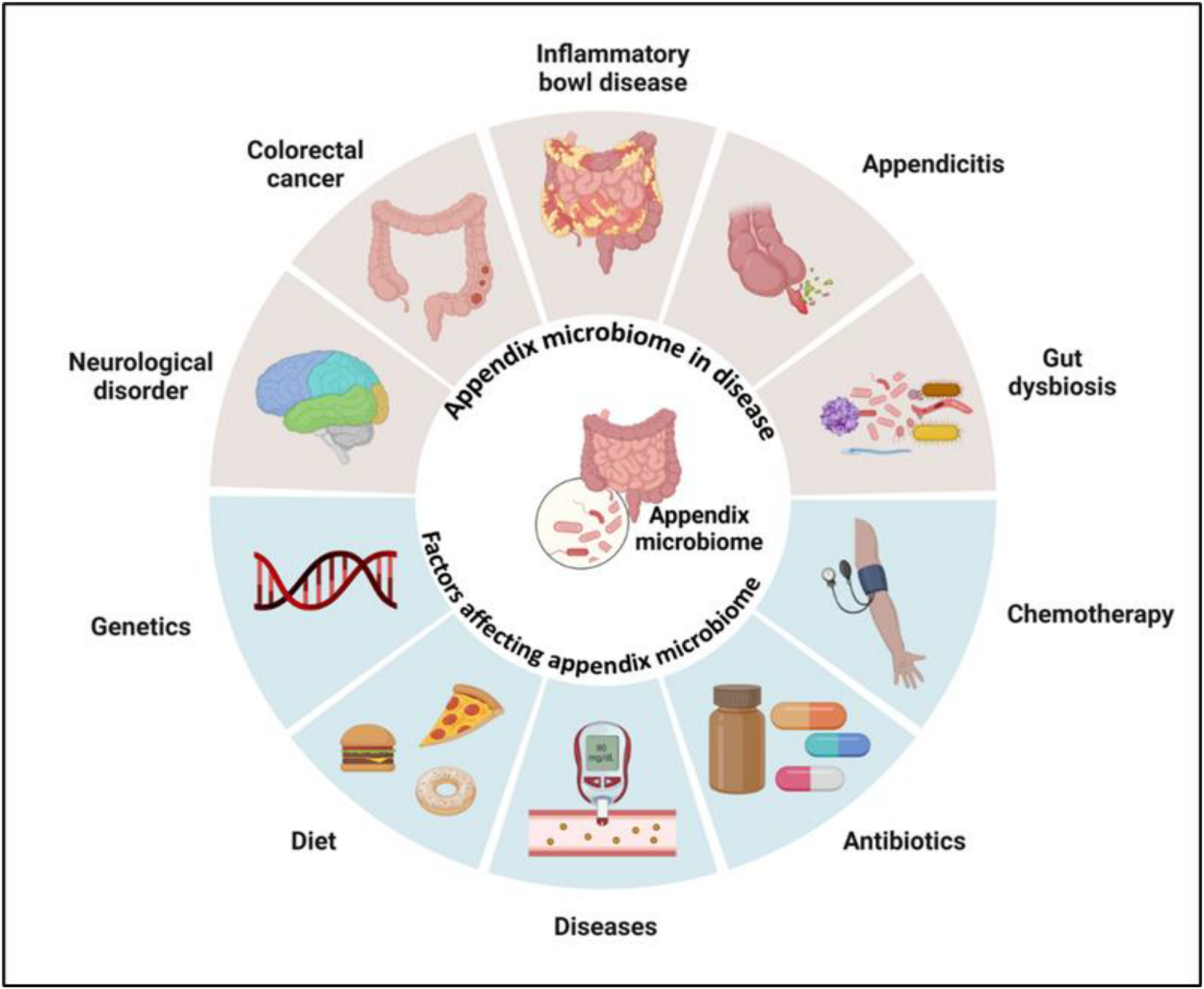
Factors influencing am composition and disease consequences caused by appendix dysbiosis. The diagram illustrates the dual aspects of the appendix microbiome in disease and the factors affecting it. The lower side shows the major factors involved in gut dysbiosis, and the upper side shows pathways leading to the physiological outcomes or diseases due to gut dysbiosis

The composition of AM and its function is impacted by various factors like diet, disease, antibiotic use, and chemotherapy (Fig. 3) [35–37]. By knowing these influences, targeted interventions can be developed, such as dietary modifications or probiotic supplementation, to maintain or restore a healthy AM [24, 38]. As research in this field advances, it will provide further insights into the complex interactions between our lifestyle, health, and the microbial communities within us.

### Effect of diet

Diet is known to be one of the decisive inputs that shape the constituents of the AM. A diet rich in fibers, for instance, increases *Firmicutes* and *Bacteroidetes*, which are beneficial and ferment dietary fibers and produce SCFAs (short chain fatty acids), crucial for colon health [35, 39]. In the gut, the fermentation of prebiotic carbohydrates like inulin and fructo-oligosaccharides promotes the growth of good bacteria, primarily *Bifidobacterium* and *Lactobacillus* species [40]. Conversely, diets high in processed and sugary foods may encourage the growth of harmful bacteria including *Clostridium innocuum, Catenibacterium mitsuokai, Enterococcus* spp. [41] *C. difficile* [42] *and C. perfringens* [43]*,* etc., leading to dysbiosis [44, 45]. A Western-style diet for one month was found to cause a 71% increase in endotoxemia. By contrast, a more health-conscious diet lead to a 31% decrease [40]. Drug taking can significantly influence the diversity of AM. GM of patients with alcoholism was significantly altered vs. sober ones. A study suggests that alcoholism significantly increases the presence of *Firmicutes* and lowers the abundance of *Bacteroidetes* [46]. Vitamins B help the proliferation and metabolic reactions of different bacteria. Around 20% gut bacteria produce Vitamin B12 and around 80% of gut bacteria, like *Pseudomonas denitrificans*, *B. megaterium*, and *Propionibacterium freudenreichi*, *B. fragilis*, *P. copri*, *C. difficile*, *F. prausnitzii*, *R. lactaris*, *B. animalis*, *B.infantis*, *B.longum*, and *F. varium*, need vitamin B12 for their metabolic reactions [47, 48]. The decreased proliferation of *Prevotella* spp., which breaks down plant polysaccharides and produces thiamine, may be caused by a lack of vitamin B1 [47]. A diet devoid of propionic acid, or taking antibiotics which reduce bacteria generating that compound was found to improve the condition of patients with autism [49]. According to recent findings, a ketogenic diet balances out an unbalanced GM. Following a ketogenic diet, there was a rise in *Bacteroidetes* and a decrease in *Proteobacteria* at the phylum level. Following the administration of a ketogenic diet for a few days, a drop in *Cronobacter* was found. Also, an increase in *Prevotella*, *Bifidobacterium*, and *Bacteroides* was observed at the genus level [50].

### Effect of diseases

Diseases, particularly those affecting the GI tract, can significantly alter the AM. For example, acute appendicitis is accompanied by a reduction in microbial diversity and an surge in potentially pathogenic bacteria like *Parvimonas* and *Acinetobacter*, which may contribute to the inflammation characteristics of the condition [36]. IBDs (inflammatory bowel diseases) have also been linked to changes in the AM, potentially affecting disease progression and outcomes [51–53]. Examples are ulcerative colitis (UC) and Crohn’s disease (CD).

### Effect of antibiotics

Antibiotic use is known to disrupt the GM, and the AM is no exception. Antibiotics can reduce the diversity of bacterial species, alter their activity and metabolism, and select antibiotic-resistant ones [54–57]. For instance, Penicillin can reduce the number of *Lactobacilli*, *Eubacteria*, *Bifidobacteria,* etc., in the gut. Similarly, quinolones (broad-spectrum bacteriocidals) can reduce the number of *Enterobacteriaceae*, *Coprococcus*, and *Lachnospiraceae* (Table 1) [58, 59]. These changes can lead to antibiotic-associated diarrhea and increase the risk of recurrent *Clostridioides difficile* infections, highlighting the importance of judicious antibiotic use to preserve the health of the AM [60]. Overuse of antibiotics can lead to the overabundance of *Desulfovibrio*, or common therapy-resistant bacteria, which emit lipopolysaccharides (LPS) [61, 62]. Behaviors resembling ASD (Autism Spectrum Disorder) were developed as a result of fetal exposure to LPS [63].

**Table 1:** Effects of various antibiotics on the abundance of AM and/or GM

Antibiotic groups	Antibiotics’ name	Microbes decreased	Microbes increased
Carbapenems	Carbapenem Meropenem Ertapenem	*Enterobacteria* *Streptococci* *Clostridia* *Bacteroides* *Bifidobacteria* *Lactobacillus* *Eubacteria*	*Enterococci*
Penicillin	Amoxicillin Clarithromycin Erythromycin Spiramycin Pen V	*Lactobacilli* Gram + ve aerobic cocci *Bifidobacteria* *Lanchnospiraceae* *Eubacteria*	*Enterobacteria* *Bacteroidaceae*
Lincomycin	Clindamycin	*Bacteroids* *Blautia*	*Enterobacteriaceae*
Cephalosporins	Cefelor Cefotaxime Ceftizidine Cefuroxime Cefepime	*Bacteroides* *Bifidobacteria* *E. coli*	*Enterobacteriaceae* *Clostridia*
Macrolides	Azithromycin Clarithromycin Erythromycin Spiramycin	*Actinobacteria* *Lanchnospiraceae* *Veillonella* *Clostrialis* *Erysipelotrichaceae*	*Bacteroides Proteobacteria* *Resistant Enterobacteria* *Streptococci* *Enterococci*
Quinolones & Flouroquinolones	Ciprofloxacin Norfloxacin	*Lachnospiraceae* *Coprococcus* *Enterobacteriaceae* Gram -ve aerobes	Resistant *E. coli*

### Effect of chemotherapy

Chemotherapy, while essential for cancer treatment, can modulate the AM [64]. Chemotherapy reduces both the number and the diversity of bacteria in the gastrointestinal tract and the feces [65]. Previous studies suggest that it can increase *Firmicutes* and reduce *Bacteroidetes* [66, 67]. Some studies suggest that chemotherapy may increase the count of different gram-negative pathogenic bacteria, like *E. coli* and *Pseudomonas,* and reduce the abundance of different beneficial gram-positive bacteria, like *Bifidobacterium* and *Lactobacillus* [65, 67–69]. In addition, this modulation is associated with a higherrisk for infections and can impact the effectiveness of the treatment. Youssef et al. reported that chemotherapy and/or radiotherapy significantly increase *Lactobacillaceae* and *Lactobacillus*, and lower *Bifidobacteriaceae*, *Ruminiclostridium*, *Lachnoclostridium*, and *Oscillibacter* [70]. Moreover, *Prevotella copri* and *Bacteroides plebeius* were higher in patients under chemotherapy [71]. This dysbiosis consequently impacts the body in different ways [72].

### Effect of genetic conditions

Through a complex interplay of host and environmental variables, host genetic variations influence GM [73]. Certain genetic variations are accountable for preserving the gut commensal makeup [74]. Variants at the LCT locus associated with higher levels of *Bifidobacterium* genus and *Negativibacillus* genus, UBA3855 sp900316885 and CAG-81 sp000435795. In addition, higher levels of *Faecalicatena lactaris* are associated with ABO variables. MED13L variants linked to colorectal cancer (CRC) showed higher levels of *Enterococcus faecalis* [75]. Similarly, PLD1 & LINGO2 variants related to obesity showed a higher number of *Akkermansia* and *Blautia* spp. [76], and NOD2 (nucleotide-binding oligomerization domain-2) variants having IBD showed a higher number of *Escherichia* spp. and a lower number of *Faecalibacterium* spp. [77]. SLIT3 (slit guidance ligand 3) variants associated with inflammation and obesity showed an increase of *Clostridiaceae* and *Dermococcus* spp. [78]. There are some more genes that impact the gut microbial composition. All of these prove a strong association of host genetics with GM.

## Appendix and/or gut microbiome in disease development

**Fig. 4:**
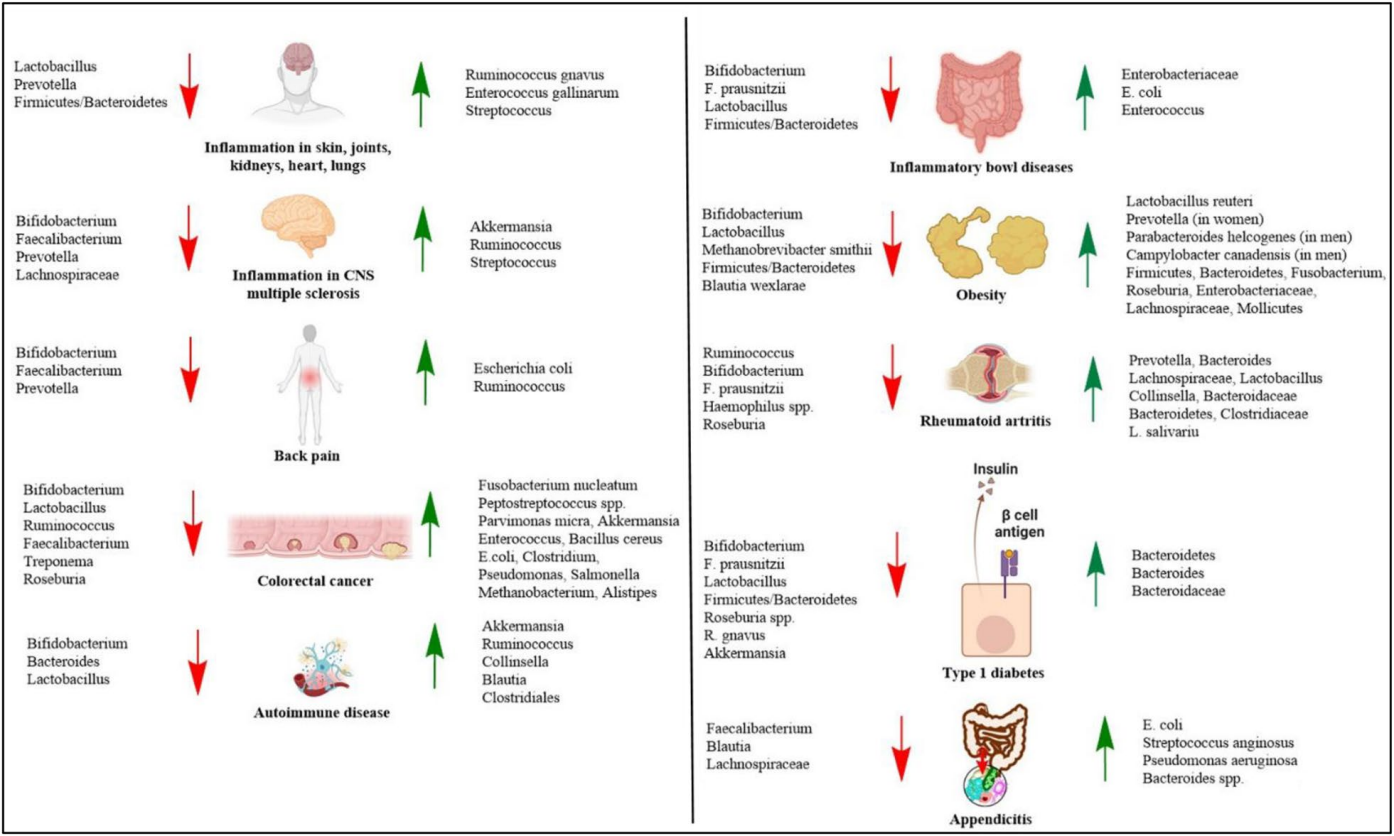
Involvement of GM in different disease development. The red arrows on the left side indicate the reduction of microbes associated with the disease development and the green arrows on the right side indicate the increase of the microbes associated with the disease development. It is clear from the figure that a reduction in the number of *Bifidobacterium*, *Lactobacillus*, *Prevotella*, *Faecalibacterium*, and *Firmicutes*/*Bacteroidetes* is associated with most of the disease progression. On the other hand, an increase in the number of *E. coli*, *Enterococcus*, *Akkermansia*, *Enterobacteriaceae*, *Ruminococcus*, *Clostridium*, and some other pathogens is associated with most of the disease progression. This could be a piece of important information for microbe-mediated therapeutics

**Fig. 5:**
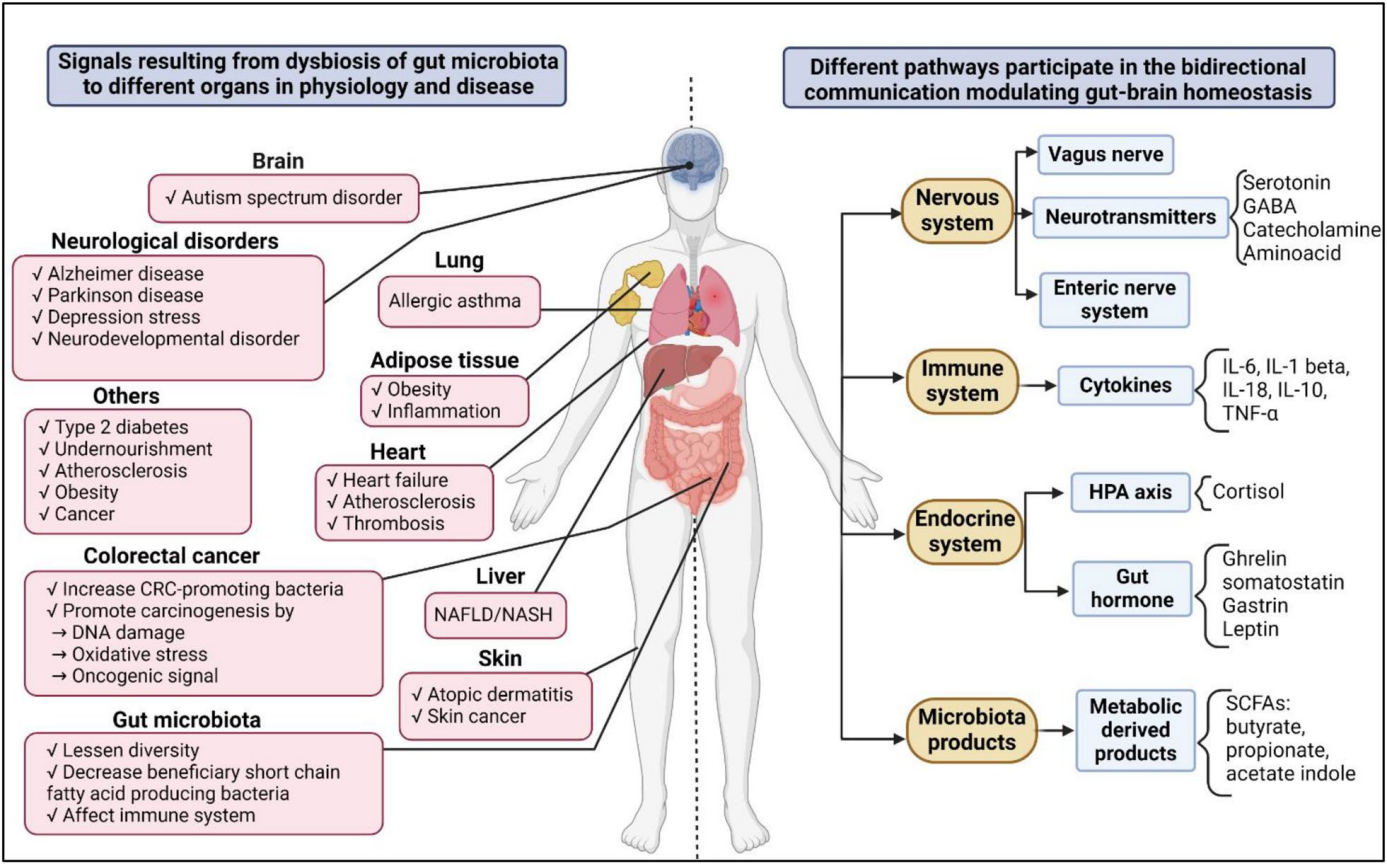
Effects of gut dysbiosis in different physiological outcomes. The left side of the figure shows the effects on different organs resulting from the dysbiosis of GM. The right side of the figure shows the pathways along the body that are involved in different physiological outcomes or diseases due to the dysbiosis of GM. *GABA* gamma-aminobutyric acid, *IL* interleukin, *TNF* tumor necrosis factor, *HPA* hypothalamic–pituitary–adrenal axis

The AM is highly important both for gut health and immune function. Changes in the AM can lead to diseases such as acute appendicitis, IBD, back pain, obesity, diabetes, cancer, and so on [52, 79, 80]. Figure 4 shows the microbial compositional changes that cause different disease development, whereas Fig. 5 shows the effects of gut dysbiosis in different physiological outcomes.

### Acute appendicitis

Globally, acute appendicitis ranks top among surgical emergencies. The conventional theory for its pathogenesis has been luminal obstruction, but emerging evidence suggests a strong involvement by the microbiome in the development of this condition [6]. *E. coli*, *Streptococcus anginosus*, *P. aeruginso*, *Bacteroides* spp., and *Klebsiella* spp. are common bacteria that can cause acute appendicitis [81, 82]. Studies have shown that patients with acute appendicitis exhibit a significant decrease in microbial diversity compared to healthy individuals. *Parvimonas* and *Acinetobacter* are increased, while*Faecalibacterium*, *Blautia*, and *Lachnospiraceae (*commensal taxa*)* [6] are reduced. *Fusobacterium* spp., *Porphyromonas,* and *Parvimonas* can be translocated from the mouth to the appendix, and these pathogeens can increase the severity of complicated appendicitis [83]. Other studies found a huge number of *Fusobacterium* spp. in the saliva and feces of children suffering from pediatric acute appendicitis [84, 85]. 16S rRNA gene sequencing of the appendiceal microbiome revealed that *Campylobacter jejuni* may be a significant cause of acute appendicitis [86]. These changes in the microbiome may result in inflammations, resulting in acute appendicitis.

### Inflammatory bowel diseases (IBDs)

IBDs are connected with severe inflammation of the GI tract. The appendix has been found to be involved in the pathogenesis and outcomes of IBDs [87, 88]. Alterations in the GM, such as decreasing the number of *Actinobacteria*, *Firmicutes*, and *Bacteroidetes*, and enrichment of *Proteobacteria*, could start the disease [79, 88]. The study revealed that patients with BD had higher concentrations of *Bilophila* spp., a sulfate-reducing bacteria (SRB), and several opportunistic pathogens, such as *Parabacteroides* spp. and *Paraprevotella* spp. Additionally, there was a lower concentration of methanogens, such as *Methanoculleus* spp. and *Methanomethylophilus* spp., and butyrate-producing bacteria (BPB) *Clostridium* spp. [89]. Recent research has highlighted significant microbiome alterations in Crohn’s patients, with the detection of the *Staphylococcus sciuri* pathogen and elevated NLRP3 protein expression, suggesting its potential as a diagnostic biomarker [90]. For instance, inflammation around the appendix, known as the “peri-appendicular patch”, has been frequently observed in patients with UC, even preceding the onset of the disease [88]. Moreover, the temperate phage viruses residing in the bacterial population display a shift from lysogenic to lytic replication in IBD patients [91]. Alterations of fungal and protozoa populations were also observed with IBD. Patients with IBD had a higher frequency of fungi, mostly *Saccharomyces cerevisiae*, and a lower frequency of protozoa, mostly *Blastocystis* species (subtypes 1, 2, 3, and 4) [92].

### Auto-immune disease

**Fig. 6:**
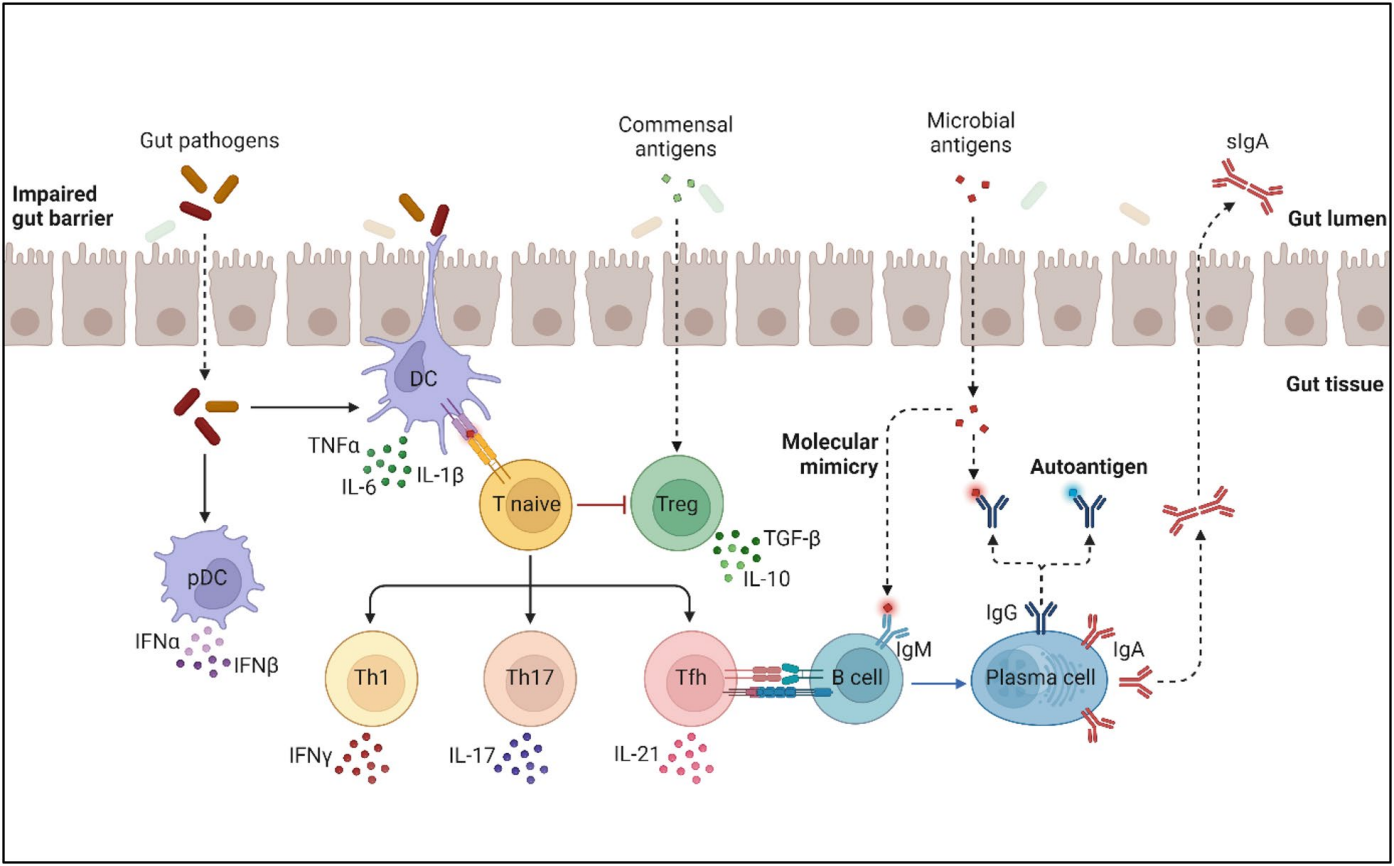
Role of microorganisms in autoimmune disease. The diagram illustrates the interactions between gut pathogens, commensal antigens, microbial antigens, and immune cells, highlighting the complex pathways involved in gut immunity and potential therapeutic targets in gut-related diseases

Gut dysbiosis has a strong relationship to the development of ADs like RA, type 1 diabetes, systemic lupus erythematosus, multiple sclerosis, spondyloarthritis, and irritable bowel syndrome [80]. The abnormal generation of autoantibodies is a characteristic of autoimmune disorders. The immune system is influenced by genetic and/or environmental variables, which result in aberrant production of proinflammatory cytokines and the decline of anti-inflammatory cytokines, autoreactive T cells, and B cells that produce autoantibodies. The rising prevalence of autoimmune disorders may be caused by significant changes in the GM as a result of dietary modifications, compromised gut integrity, extensive use of antibiotics, and other factors [68, 80, 93]. Molecular mimicry, effects on intestinal mucosa permeability, the host immunological response triggered by the microbiota, antigenic mimicry, and epitope spreading are among the potential pathways leading to AD (Fig. 6).

An important player in the immune response is the aryl hydrocarbon receptor AhR, a ligand-activated transcription factor that detects substances found in food, secondary metabolites produced by microbes, and environmental contaminants to regulate transcriptional programs in the immune system. Gut barrier development and maintenance depend heavily on AhR [94]. AhR may be involved in autoimmune illnesses through the translation of extrinsic and intrinsic signals into cellular responses through its binding to a variety of ligands coming from diet, cells, and microbes [95]. In AhR-deficient animals, reduced levels of innate IL-22 facilitated the growth of immunological activator commensal segmented filamentous bacteria (SFB), which in turn promoted Th17 cell proliferation. By inhibiting pathogenic Th17 cells, the innate expression of AhR reduced the impact [96]. The AhR ligand 2,3,7,8-tetrachloro dibenzo-p-dioxin (TCDD) caused changes in the abundances of SFB and the immunological suppressor *Bacteroides fragilis* in mice. Furthermore, the GM's presence of SFB considerably controlled the TCDD-induced host response, indicating the therapeutic potential of AhR ligands and important commensals [97].

Another cause of autoimmune illness is dysbiosis. The GMs molecularly mediate autoimmune through cross-reactivity and posttranslational modification of autoantigens. Live gut bacteria can translocate across a malfunctioning gut barrier, causing direct interactions with tissue and immune cells that in turn trigger systemic autoimmunity at the cellular level [98]. Research conducted both in vitro and in vivo has demonstrated that dysbiosis in patients with systemic lupus erythematosus (SLE) can enhance Th17 differentiation and lymphocyte activation. Conversely, *Bifidobacterium bifidum* supplementation can balance the Treg/Th17/Th1 ratio and prevent CD4 + lymphocytes from being overactivated [99]. Additionally, serum antibodies against *Ruminococcus* antigens and decreased bacterial diversity with a five-fold increase in *R. gnavus* prevalence in SLE patients point to the role of GM dysbiosis in the pathophysiology of lupus nephritis [100]. Similarly, autoantibody levels and the duration of RA were linked to low gut microbial diversity and high *Collinsella* abundance, which was coupled with the pro-inflammatory cytokine IL-17A. This shows that these variables may be used to predict the status of RA disease [101]. Additionally, compared to ACPA-negative persons, RA patients with positive tests for the anti-citrullinated protein antibody (ACPA) showed a lower level of microbial diversity and enrichment of *Blautia*, *Akkermansia*, and *Clostridiales* [102].

There are a number of potential explanations for the correlation between dysbiosis of the GM and autoimmune disorders, including effects on the functioning of the human immune system. For example, altering the host immune response and stimulating antigen-presenting cells (APCs), such as dendritic cells (DCs), may cause antigen presentation and the release of cytokines, which in turn may impact the development and functionality of T cells. Moreover, this effect throws off the balance between T regulatory cells (Tregs) and T helper 17 (Th17) cells. Antigenic mimicry has been linked to similarities between foreign and self-antigens; as a result, pathogen-derived autoreactive T and B cells are activated, which in turn promotes autoimmunity [93]. Some microbes can change the host antigen using their own enzyme-producing neoepitopes which are detected by the immune system causing autoimmune disease. A greater abundance of *Prevotella copri* has been linked to higher protein citrullination which subsequently stimulates pro-inflammatory cytokines, leading to damaged tissues, and self-antigens release, which supports to the development of autoimmune responses [103].

### Cancer

Appendicitis can be a precursor to appendix cancer. Tumors within the appendix can block the organ, leading to the trapping and overgrowth of bacteria normally present in the intestines. This bacterial overgrowth may contribute to inflammation and other changes that could eventually lead to cancer [104]. While R&D has concentrated on the relationship between bacteria and appendix health, there is currently limited understanding of how intestinal fungi may be involved [7, 105]. Mechanisms by which AM influences cancer risk include immune modulation, production of carcinogenic metabolites, and direct interactions with mucosal cells (Table 2) [106, 107]. Emerging evidence underscores the crucial role of gut microbes in CRC development [17, 108]. Recent studies have proposed the involvement of *Fusobacterium nucleaturm*, *Pseudomonas*, *Streptococcus bovis*, *Salmonella*, *Helicobacter pylori*, *Akkermansia*, *Bacteroides fragilis*, *Peptostreptococcus,* and *Clostridium septicum*, in the CRC progression [80, 109]. Metabolites of these bacteria like bile salts, hydrogen sulfide, and trimethylamine-N-oxide (TMAO) cause inflammation, DNA damage, and subsequently cancer development [109]. Observational studies and clinical trials suggest an association between GM and cancer. Recent studies, including a two-sample Mendelian randomization study, have investigated the causal link between GM and cancer. These studies identified multiple associations between genetic liability in the GM including the genus *Bifidobacterium* and cancer, emphasizing the microbiota's causal role in cancer development [110, 111]. Pancreatic ductal adenocarcinoma (PDAC) is one of the fatal solid tumors which is resistant to immunotherapy. Metagenome analysis of the AM of PDAC patients showed an abundance of *Klebsiella pneumoniae*, *Bifidobacterium animalis*, and *Adlercreutzia equolifaciens*, while certain commensals are depleted [112]. Additionally, identifying microbial signatures associated with cancer may lead to novel biomarkers for early detection [113, 114].

**Table 2:** Gut-microbial involvement in different types of cancer progression

Domain	Microorganisms	Metabolites	Cancer type	Mode of action	References
Bacteria	*Fusobacterium nucleatum*	Fusobacterium adhesion protein (FadA)	CRC (colorectal)	Promotes adhesion and invasion of cancerous cells	[115, 116]
	*E. coli*	Colibactin, Cytolethal distending toxin (CDT), Genotoxin,	Various cancers (including colorectal, breast, and pancreatic)	Genotoxicity. Disrupts cell cycle and promotes DNA damage	[117, 118]
	*Peptostreptococcus spp.*	Hydrogen sulfide	CRC	Promotes inflammation and genotoxicity	[119]
	*Helicobacter pylori*	Cytotoxin-associated gene A (CagA)	Gastric Cancer	Chronic inflammation induced by the bacteria can damage the stomach lining	[116, 120, 121]
	*Parvimonas micra*	LPS	CRC	May induce chronic inflammation and immune suppression	[122, 123]
	*Enterococcus faecalis*	Unidentified	CRC	Increase the frequency of aneuploidy and tetraploidy	[124]
	Sulfidogenic Bacteria (i.e. *Fusobacterium*, *Desulfovibrio, Bilophila wadsworthia)*	Hydrogen sulfide (H_2_S)	CRC	DNA damage, Effect DNA repair, Chromosomal instability (CIN), and Interference with mitochondrial function	[116]
	*Bacteroides fragilis*	*B. fragilis* toxin (BFT)	CRC	May contribute to tumor growth and metastasis	[125]
	*Akkermansia muciniphila* (Possible protective effect)	Short-chain fatty acids (SCFAs)	Various cancers	May enhance anti-tumor immunity and reduce inflammation	[126]
Fungi	*Candida albicans*		Various GI cancers	May promote inflammation, reduce cell adhesion (facilitating metastasis), and influence tumor gene expression	[127]
	*Malassezia spp.*	Possibly unidentified metabolites	Breast cancer	Potential role in promoting inflammation and reducing survival rates. More research is needed	[128]
Archaea	*Methanobrevibacter spp.*	Methane	CRC	Disruption of gut barrier function and inflammation	[129]
	*Methanothermobacter spp.*		CRC (indirect—via IBD)	Modulation of gut immune response (linked to IBD)	[130]
	*Thermoplasmatales*		CRC	Unclear, potentially linked to gut inflammation	[131]
	*Candidatus Mancarchaeum acidiphilum*		CRC (potential biomarker)	Correlated with other cancer-associated microbes, function unknown	[131]

### Obesity

An increasing amount of data is available on modifications to microbiota and obesity, along with the illnesses linked to it. Age-related, exercise-related, and weight-related alterations have all been documented [132]. The intestinal microbiota influences the development of obesity [133, 134].

Conversely, individuals with high blood pressure and resistance to insulin had lower bacterial richness. Additionally, distinct correlations were discovered between elevated pressure and the existence of *Clostridium* and *Clostridiaceae*, reduced low-density lipoprotein (LDL) and *Lachnospiraceae*, *Gemellaceae*, *Turicibacter*, and elevated risk of metabolic syndrome [135]. Similarly, intestinal abundance of *Akkermansia muciniphila* has been observed to be negatively correlated with the probability of greater severity of insulin resistance in both lean and obese Asian patients [136].

The effects of GM on other metabolic processes linked to immune functions may also have an impact on bodyweight control. This is primarily because GM has been linked to elevated levels of bacterial LPS, which have been found in subjects whose obesity was brought on by a high-fat diet [137]. Increased intestinal porosity, an immunological response in the mucosa, and increased intestinal permeability are all caused by dysbiosis. It seems that the microbiota facilitates the transfer of LPS but also releases it, which might result in insulin blockage and metabolic endotoxemia [132, 137]. Moreover, research shows the ratio of *Firmicutes*/*Bacteroides* in the obese gut [138]. In another study, infants with normal weight showed a higher richness of *Lactobacillus*, whereas *Rhomboutsia* and *Clostridium *sensu stricto predominated in the obese cohort [135].

### Diabetes

An autoimmune response is thought to cause Type 1 diabetes. The beta cells in the pancreas, which produce insulin, are destroyed by this process. Type 1 diabetes patients were found with a reduced abundance of *Bifidobacterium*, *Lactobacillus*, *Faecalibacterium*, *Firmicutes*/*Bacteroidetes* ratio, and a greater abundance of *Bacteroides* and *Bacteroidetes* [80]*.* Another study on gnotobiotic mice (born in germ-free conditions and inoculated with known bacteria) discovered that the intestinal bacteria impacted glucose metabolism raised the accumulation of macrophages toward the pro-inflammatory M1 phenotype in white adipose tissue, and ultimately led to the formation of insulin resistance. This finding established a link between bacterial LPS and insulin resistance [139]. The existence of *Coriobacteriaceae* (*Faecalibacterium prausnitzii)*, *Parabacterodes*, *Bacteroides caccae*, *Oscillospira*, *Parabacteroides distasonis*, *Coprococcus*, and *Haemophilus parainfluenzae* was reported to be linked to a reduced metabolic syndrome score, fasting glucose, and HOMA_IR (Homeostatic Model Assessment for Insulin Resistance) [135].

### Rheumatoid arthritis

It has been shown that the bacterial makeup of the intestinal tract differs in patients with rheumatoid arthritis (RA) compared to healthy individuals. Reduced numbers of *Firmicutes*, *Bacteroides*, *Bifidobacterium*, *F. prausnitzii*, *Haemophilus* spp., and *Roseburia* were observed in RA patients, while increased numbers of *Prevotella copri*, *P. gingivalis*, *Collinsella*, *Lactobacillus*, *Bacteroidaceae*, *Lachnospiraceae*, *Bacteroidetes*, *Clostridiaceae*, and *L. salivarius* were observed [80, 140–142]. RA caused by GM is mainly a result of an autoimmune response [140, 142]. Dysbiosis triggers autoreactive T cells in the colon, which enhances susceptibility to arthritis. Dysbiotic microbiota in the colon trigger autoreactive SKG mice T cells, which results in inflammation of the joints [142]. *P. copri* dominated the GM of patients with early-stage RA. When given zymosan, SKG mice with microbiota from RA patients experienced severe arthritis and an increase in intestinal Th17 cells [140, 142]. In individuals with new-onset untreated rheumatoid arthritis (NORA), increases in *Prevotella* abundance were associated with decreases in *Bacteroides* and a loss of microorganisms that were thought to be helpful. An elevated susceptibility to chemically induced colitis was another effect of *P. copri* [103].

## Role of appendix and/or gut microbiome in neurological disorders

The GBA is an intricate bidirectional communication network that links the GI tract with the central nervous system (CNS). This dynamic interplay involves complex interactions through neural, immune, and endocrine pathways [143, 144].

The GM contributes to CNS homeostasis by modulating the immune system and regulating the production of various molecules and metabolites [38, 145]. These metabolites, such as SCFAs produced by *Bacteroidetes* and *Firmicutes*, neurotransmitters, and other signaling molecules, can cross the blood–brain barrier and impact brain function directly [146, 147]. Moreover, the immune system's interaction with the GM helps maintain a balanced inflammatory response, which is crucial for preventing neuroinflammation [148, 149].

Neurological disorders (NDs) such as depression, schizophrenia, Parkinson disease (PD), Amyotrophic lateral sclerosis (ALS), autism spectrum disorder (ASD), epilepsy, and migraine have been linked to dysbiosis in GM [150]. While working with the C57BL/6 J mouse model, depletion of *Lactobacillus* and enrichment of *Akkermansia* were associated with neuroinflammation [151]. According to a recent study, stress may have caused gut dysbiosis, which in turn may have increased the abundance of conditional pathogens like *Enterococcus*, *Vagococcus*, and *Aerococcus*, as well as pathogens like *E. coli* and *Shigella* [152]. Alcoholism patients' GM differed significantly from healthy adult patients' GM. *Firmicutes* greatly increased in number whereas *Bacteroidetes* significantly declined. Mice receiving fecal microbiota from alcoholism patients displayed behaviors resembling anxiety and depression, a reduction in socialization, a spontaneous favor for alcohol, and a reduction in brain-derived neurotrophic factor (BDNF) when compared to mice transplanted with fecal microbiota from healthy male adults [46]. It's interesting to note that individuals with schizophrenia typically experience GI complications, have a greater frequency of intestinal barrier failure, and exhibit higher bacterial translocation [153]. The genera like *Succinivibrio*, *Megasphaera*, *Collinsella*, *Clostridium*, *Klebsiella*, and *Methanobrevibacter* were significantly higher in schizophrenia, whereas *Blautia*, *Coprococcus*, and *Roseburia* were abundance in healthy controls. Patients with first experience of schizophrenia significantly lowered numbers of *Bifidobacterium*, *E. coli*, and *Lactobacillus* [154]. A similar result was reported by other studies that showed a correlation between *Succinivibrio* and *Corynebacterium* and the degree of schizophrenia symptoms [155]. Another study reported an increase in *Anaerococcus* and a decrease in *Haemophilus*, *Sutterella*, and *Clostridium* in schizophrenia [156].

PD showsdegeneration of dopaminergic neurons. GM alterations may precede the onset of motor symptoms in PD. Interventions aimed at restoring microbial balance could potentially slow down disease progression [157, 158]. Increased genus *Akkermansia*, which promotes intestinal permeability, and decreased SCFA-producing bacteria of the genera *Roseburia* and *Faecalibacterium* are frequently linked to PD [159]. The most consistent change in the GM composition in PD was found in a more recent meta-analysis of eleven case–control studies, which revealed decreased genus *Faecalibacterium* and *Lachnospiraceae* family and increased genera *Lactobacillus*, *Akkermansia*, and *Bifidobacterium* [160]. Shen et al. reported similar changes in a meta-analysis involving fourteen studies. Reductions in *Prevotellaceae*, *Faecalibacterium*, and *Lachnospiraceae* were observed in the analysis, whereas increases in *Bifidobacteriaceae*, *Ruminococcaceae*, *Verrucomicrobiaceae*, and *Christensenellaceae* were noted [161]. Rodent models of PD have shown a similar pattern of gut dysbiosis [162]. Conflicting findings regarding the role of the appendix and PD been reported by recent studies. Park et al. found no link between appendectomy and risk change of PD in the Korean population, indicating that appendectomy did not determine the risk of PD [163]. In contrast, Wang et al. found out that appendectomy is associated with a lowered risk of PD, highlighting a potential protective effect of the surgical removal of the appendix [164]. Similarly, Jain et al. found no increased risk of PD following appendectomy, suggesting that the appendix is not a critical site for the development of PD pathology [165].Nakahara et al. described a significant phylogenetic difference in the gut microbiota between PD patients and healthy controls who had undergone appendectomy [166]. Their findings suggested a potential role of the gut microbiota, in particular the family Enterobacteriaceae, in the development of PD. The variable findings of these studies point to a variety of limitations and areas for future research. The studies were conducted in different populations, and this may be the reason behind the variable results. The studies were conducted through different methods, such as Cox regression analyses and Mendelian randomization [163, 164, 166], which may influence the results. Genetic susceptibility to PD may vary between different populations, and this might influence the relationship between appendectomy and risk for PD. Potential confounders such as age, sex, and underlying illness may not have been accounted for in all of the studies. Larger populations and more representative groups are needed to validate the results and a clearer causal link. Some studies, like Nakahara et al. are preliminary and must be validated by larger, more precise studies [166]. To bridge these gaps, subsequent studies should involve large-scale, multi-center studies with heterogeneous populations to account for both genetic as well as environmental heterogeneity. Standardized methods between studies would allow better comparison of outcomes. In addition, control of potential confounders is important to differentiate the true effect of appendectomy on the risk of PD. Long-term follow-up studies are required to ascertain the timing of the relationship between appendectomy and pathogenesis of PD. Mechanistic investigations should also study the precise mechanisms by which appendix and gut microbiota affect PD pathology.

ALS involves the degeneration of motor neurons. While less is known about the GM's role in ALS compared to AD and PD [167]. A study with ALS SOD1G93A mice yielded a reduction in *Butyrivibrio fibrisolvens* [162], which liberate butyrate. However, while working with humans no substantial changes were found in the ratio of *Firmicutes*:*Bacteroidetes* [168].

ASD is a collection of neurodevelopmental disorders. Concomitant pathologies in patients with ASD typically include gut dysbiosis, anxiety, depression, seizures, and other GI issues, like diarrhea, abdominal discomfort, and vomiting [169, 170]. It has been observed that people with ASD have higher biomass and less variation in bacteria [171, 172]. The most significant might be lower concentrations of *Bacteroidetes*, *Actinobacteria*, *Proteobacteria*, *Prevotella*, *Corprococcus*, and *Veilonellaceae* [171], combined with higher concentrations of *Desulfovibrio* spp., *Sutterella* spp., and *Ruminococcus torques*, or *Clostridium* spp. [173, 174]. Additionally, other authors reported an excess of *Megamonas*, and *Candida* spp., which were found to set freeammonia and other toxins that contribute to autistic behavior. They also saw a decline in the ratio *Bacteroidetes*:*Firmicutes* [174–176]. Additionally, the onset of Asperger's syndrome was connected with a decrease of pro-inflammatory in *Bifidobacterium* spp. [177]. According to earlier research, patients with autism may benefit from butyrate-producing bacteria like *Eubacterium*, *Ruminococcaceae*, *Erysipelotrichaceae*, and *Lachnospiraceae* [178].

Epilepsy is a seizure disorder and aneurological disease. GM composition differs between drug-resistance and drug-responsive epilepsy patients. Compared to patients who experienced more than four seizures annually, those who experienced four or fewer per year displayed higher levels of *Bifidobacteria* and *Lactobacillus* [179]. Recent studies suggest a ketogenic diet to restore GM and protest epilepsy. The level of *Parabacteroides* and *Akkermansia muciniphila* was elevated by the ketogenic diet. Seizures were prevented when gnotobiotic cocolonization was enhanced with *Akkermansia* and *Parabacteroides* [180]. Moreover, probiotic supplementation of *Lectobacillus* and *Befidobacterium* reduced 50% of drug-resistance epilepsy [181].

A “leaky gut”-related increase in proinflammatory cytokines may contribute to the onset of migraine discomfort [182]. A significant correlation between GM and migraine was established. There was a notable enrichment of *Firmicutes*, particularly *Clostridium* spp., in the migraine group, whereas, the healthy controls had higher concentrations of advantageous bacteria, including *Methanobrevibacter smithii*, *Bifidobacterium adolescentis*, and *Faecalibacterium prausnitzii* [183].

## Role of appendix and/ or gut microbiome in parasitological disorders

The microbiome in the gut plays a very crucial role in protecting the host organism against numerous infections, including those caused by parasitic protozoa and fungi. The intestinal microbes regulate fat storage, stimulate epithelial cells, and influence brain and immune system development. All these interactions are significant in maintaining homeostasis and preventing infections [184, 185]. Intestinal protozoa-microbiome interactions emphasize the immunomodulatory effect of the intestinal microbiota on protozoan infection like *Entamoeba histolytica*, *Blastocystis* spp., *Giardia duodenalis*, *Toxoplasma gondii*, and *Cryptosporidium parvum* [184, 185]. The organization of intestinal bacterial communities can modulate the path of protozoan infection and the destiny of parasitic disease [186]. In parasitic illnesses associated with intestinal parasites, e.g. amoebic colitis or amoebic liver abscess due to *E. histolytica* or intestinal giardiasis due to G. duodenalis, interactions between intestinal bacteria and such parasites could affect parasitic or bacterial virulence. For instance, reduced abundance of Bacteroides, Clostridium, and Lactobacillus and increased abundance of Bifidobacterium is associated with amoebic colitis, while increased abundance of *Prevotella copri* is associated with amoebic diarrhea [187, 188]. Non-pathogenic parasitic protozoa also have the potential to alter intestinal microbiota diversity and provoke host immune responses. For example, asymptomatic colonization with *Blastocystis* spp. is associated with raised total diversity of intestinal bacteria and widespread changes of the dominant species within the gut microbiota. But colonization by *Blastocystis hominis* has been associated with reduced numbers of some markers of intestinal immunity, suggesting an anti-inflammatory environment within the intestine [184, 189, 190].

Current investigations explore the potential of fecal microbiota transplantation (FMT) to control immune response. There is evidence that FMT from protozoa-exposed donors can inhibit the immune response in a germ-free mouse model, demonstrating its potential to modulate immune responses and influence intestinal physiology [184, 188]. Specifically, gut protozoa such as Blastocystis hominis and Entamoeba histolytica can modulate the immunoinflammatory status and induce functional changes in the intestine by interacting with the gut microbiota [188, 191]. This downregulation of intestinal immune response includes decreased production of such proinflammatory cytokines as IL-6, TNF, IFN-γ, MCP-1, IL-10, and IL-12 in FMT-receiving mice from protozoa-exposed donor mice [188, 192]. Investigation into intelligent biological networks indicates a potential role through increasing anti-microbial resistance resilience by nutritional interventions. Enteric protozoan pathogenic infection effect on global health and the gut microbiome contribution towards infection is high-profile. Nutritional supplementation, including proteins, prebiotics, probiotics, synbiotics, and food formulations, is discussed as being a key part of recovery from dysbiosis and resistance to opportunistic pathogens [193]. Systems biology strategies are valuable tools to define the biochemical pathways of infection and recovery, allowing for better understanding of the impact of nutritional treatments on the gut microbiome and its role in protozoal infections [193, 194].

The imbalance of the gut microbiota, known as dysbiosis, can significantly impact the health status of the host. For example, *Cryptosporidium* spp. is an epithelial-lining-bound parasite that interacts with bacterial biofilms in the lining of the gut to cause behavioral adaptation in itself and neighboring bacterial populations [193, 195]. Studies have shown that Cryptosporidium growth is directly proportional to *Pseudomonas aeruginosa* biofilm maturation, indicating the complex interaction between parasites and bacterial communities [193, 195]. Similarly, Giardia infection may provoke alterations in gut microbiota, converting commensal bacteria into pathogenic forms and leading to increased virulence and inflammation [196]. In addition, protozoan infections can produce cross-organ effects. For instance, Cryptosporidium and Entamoeba have been known to cause opportunistic bronchial and pulmonary invasion, leading to respiratory conditions such as pneumonia, cough, and chest pain [193, 197]. Infections of the enteric type can lead to the host immune system releasing cytokines, which in turn cause inflammation and asthma. Exaggerated production of short-chain fatty acids (SCFAs) during infection potentially controls the immune system and dampens allergic responses, permitting regulatory T-cell differentiation [198]. These experiments highlight the interplay between the appendix, gut microbiota, and parasitological diseases. Microbiota manipulation has the potential to offer novel therapeutic approaches to the management of intestinal protozoan infections and related immune responses.

## Appendix microbiome as a possible therapeutic approach

**Fig. 7:**
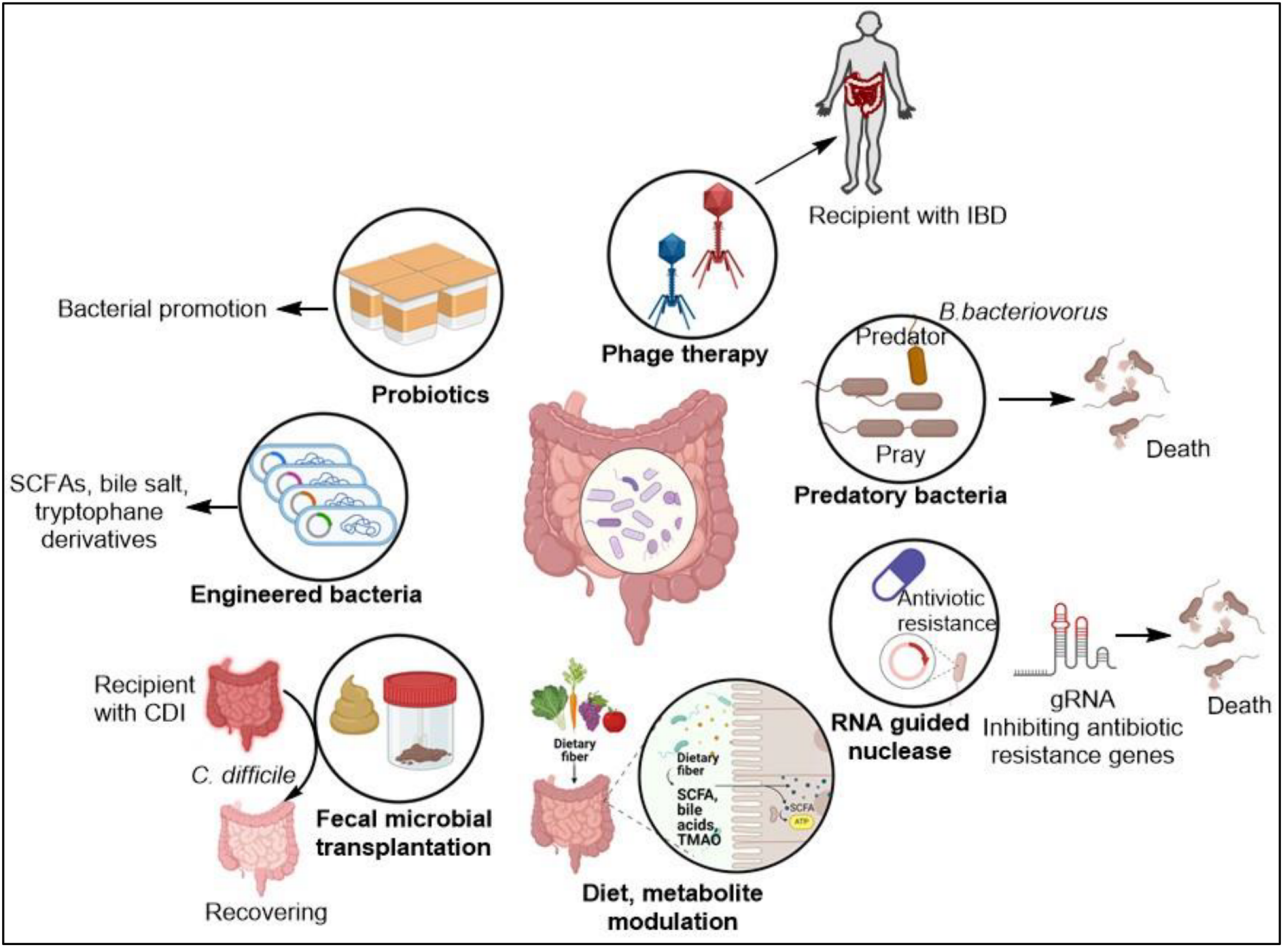
Appendix microbiome as possible therapeutic. The figure shows different possible methods for reforming gut dysbiosis, treating diseases, controlling pathogens, and inhibiting resistance or virulence genes

The AM may help to prevent and treat various diseases and could be leveraged for therapeutic approaches. They could be used to treat cancer, GI diseases, metabolic disorders, neurological disorders, cardiovascular diseases (CVDs), and so on [60, 115, 199–201]. Methods that were applied to treat these diseases using appendix/GM are the use of prebiotics, probiotics, FMT (fecal microbial transplantation), engineered bacteria, phage therapy, RNA-guided gene therapy, and diet and metabolic modification (Fig. 7) [60, 199, 202–205]. The main purpose of these methods is to reform the GM which ultimately impacts the overall health outcome.

### Prebiotics, probiotics, and postbiotics

Prebiotics are food ingredients that help the growth of helpful gut bacteria and thereby benefit the host. There are different types of prebiotics like fructans, galacto-oligosaccharides (promotes the growth of *Bifidobacteria*, *Lactobacilli*, *Enterobacteria*, *Bacteroidetes*, and *Firmicutes*), starch and glucose-derived oligosaccharides (promotes the growth of *Bifidobacteria*, and *Firmicutes*) [206]. Probiotics are beneficial bacteria that keep the gut healthy. Recent studies suggest that probiotics could serve as an adjunctive therapy in acute appendicitis, hinting at the broader therapeutic potential of targeting the AM. By influencing the immune response and possibly restoring a healthy microbial balance, probiotics might offer a non-surgical alternative to appendectomy in uncomplicated cases of appendicitis [2, 204].

Probiotics influence the efficacy and toxicity of cancer treatments [6, 115]. Probiotics, such as *Lactobacillus casei* supplements, have shown promise in reducing chemotherapy-induced diarrhea, a common side effect [207]. Over the past ten years, *Akkermansia muciniphila*, a representative commensal bacterium, has drawn a lot of interest as an effective Next-generation probiotic (NGP) that can influence tumorigenesis either directly or indirectly. It does this, in part, through its effects on antitumor immunosurveillance, which includes stimulating pattern recognition receptors (PRRs) and improving outcomes of GI cancer [208].

Probiotics also serve as a promising solution in reducing the severity of neurological disorders. Previous results suggest that treating CD with *Faecalibacterium prausnitzii* as a probiotic may be a promising approach for preventing dysbiosis [209]. In a clinical trial, patients with drug-resistant epilepsy benefited from a 4-month probiotic regimen that included *L. acidophilus*, *L. plantarum*, *L. casei*, *L. helveticus*, *L. brevis*, *B. lactis*, and *Streptococcus salivarius* subsp. *Thermophilus*. This regimen resulted in a ≥ 50% drop in the number of seizures in 28.9% of the patients [181]. A mother’s GM needs to be healthy and balanced during pregnancy to maintain a healthy and balanced milk microbiota composition [210]. Furthermore, oral probiotic administration may enhance the quantity of *Bifidobacterium* and *Lactobacilli* in breast milk in moms who give birth vaginally [210].

Postbiotics are bioactive compounds produced by probiotics upon digestion of prebiotics. Using postbiotics is a unique strategy for treating disorders like CVD and gut dysbiosis at the same time. They are described as the creation of inactive microbes and/or their components that benefit the host's health [211]. The connections between gut microbes and brain cells, such as microglia, astrocytes, and neuronal cells, are impacted differently by diet-derived gut microbial metabolites. Methods to alter microbial metabolite levels have been proposed as a target for neuropsychiatric disorders [212, 213]. Colonocytes and a number of other cell types respond positively to butyrate in vitro. Butyrate reduced the expression of vascular cell adhesion molecule-1 (VCAM-1) and intracellular cell adhesion molecule-1 (ICAM-1) caused by TNFα. IL-1-stimulated VCAM-1 and ICAM-1 expression was suppressed by butyrate at 10 mmol/L [214]. When sodium acetate (1 g/kg) is given intraperitoneally into radiotelemetry-implanted mice, acetate reduces both the heart rate (HR) and the mean arterial pressure [215]. Propionic acid, a molecule generated from microbiota, has shown remarkable efficacy in the metabolism of intestinal cholesterol. After taking 500 mg twice a day for eight weeks, hypercholesterolemic patients showed reductions in total cholesterol, very-low-density lipoprotein (VLDL), and low-density lipoprotein (LDL) [216].

### Diet and metabolites

Eating a balanced and healthy diet and its microbe-derived metabolites is an excellent solution for GM-mediated therapeutics for different diseases. In vivo, study shows that exogenous *Lactobacillus reuteri* inhibits the growth of colon tumors, increases tumor reactive oxygen species, and reduces translation of proteins. As shown by microbial profiling, *L. reuteri* and its metabolite, reuterin are downregulated in human and mouse CRC [217]. In female mice, estrogens can slow the evolution of CRC by increasing the colonic expression of SLC3A2, which binds to the D-alanyl-D-alanine carboxypeptidase (DD-CPase) of *Carnobacterium maltaromaticum* [218].

The unique bacterial populations in the appendix may influence metabolic pathways and gut hormone production. AM could regulate the production of SCFAs, which are important for insulin sensitivity and energy balance [219–221]. Certain appendix bacteria (i.e. *Proteobacteria*, *Bacteroidetes*) could produce or influence the production of neurotransmitters such as serotonin and gamma-aminobutyric acid (GABA) [222, 223]. These compounds are known to control the mood.

The GM’s impact on CVD is multifaceted, affecting systemic inflammation, cholesterol metabolism, and blood pressure all vital in CVD development [224–226]. Dysbiosis can lead to inflammation and compromised intestinal barriers, increasing CVD risk. Microbial metabolites like trimethylamine N-oxide (TMAO), linked to atherosclerosis, are produced by gut bacteria (i.e. *Acinetobacter*, *Pelobacter*) from dietary nutrients [227]. Additionally, GM (i.e. *Bacteroides* sp, *Clostridium, Eubacterium*) influences bile acid metabolism, affecting cholesterol levels and CVD risk [201, 228, 229].

### Fecal microbial transplantation (FMT)

FMT has shown promise in restoring GM balance after certain cancer treatments [202]. Additionally, the emerging field of pharmacomicrobiomics explores how GM influences an individual's response to medications. By analyzing GM, doctors may give a prediction of patient response to cancer treatments, paving the way for personalized medicine [230]. FMT has shown promise in treating IBD [231]. Therapeutically, there is potential for using appendix-derived microbiota in FMT for patients with metabolic disorders [199]. This targeted approach could improve insulin sensitivity, reduce inflammation, and promote a healthier metabolic profile [199]. Additionally, FMT is being studied for its ability to restore microbial balance and improve neurological outcomes. FMT restores a healthy microbial community in the patient’s gut, which could have positive impacts on the GBA and neurological health [232–234]. Therapeutically, there is potential for using appendix-derived microbiota in FMT for individuals with depression or anxiety [235]. This targeted approach could modulate the GBA and improve neurological health.

### Engineered microbes and gene therapy

Potential live microbial biotherapeutics are made by engineered resident gut bacteria, which also make research easier into the effects of microorganisms and their metabolites on human health. The roles of the GM and its metabolome in the gut can be investigated in a more regulated, physiological setting by altering gut bacteria [236]. Different methods used to derive the desired engineered microbe include metabolic modifications and genetic modifications by loss of or gain of function [236]. For instance, In vitro investigations by the deletion of a bile salt hydrolase in *B. thetaiotaomicron* showed that decreased body weight and alterations to the host's metabolic, transcriptional profile, and immune response pathways are caused by the manipulation of tauro-β-muricholic bile acid [237]. In a similar study, CRISPR-Cas9-induced deletions were employed in *Clostridium sporogenes* to remove pathways that generate microbial metabolites. The impact of removing particular metabolites on host physiology was then examined. When genes for 10 *C. sporogenes* were knocked out, it produced microbial products IgA–modulatory activity [238]. The genes required for the whole manufacturing pathways known as lithocholic acid (LCA) and bile acids deoxycholic acid (DCA), which are known to inhibit the growth of *Clostridium difficile* and are two of the most prevalent small molecules in the microbiome of *C*. *scindens*, accumulate at approximately 500 μM. Subsequently, the genes required for the biosynthesis of LCA and DCA were refined and introduced into the genetically tractable producer *C. sporogenes*. [239].

Engineered gut bacterial were used in different studies to restore and rebalance GM [240–242]. Irritable bowel syndrome and IBD are two GI disorders that have been treated with *E. coli* Nissle 1917 (EcN) [241, 242]. EcN is thought to prevent the formation of opportunistic infections by producing microcin proteins or siderophores that scavenge iron, which inhibits the growth of *Salmonella* spp. and other coliform enteropathogens [240, 242].

Developing a new class of engineered live bacterial therapeutics to treat human diseases is another promising advance of science [243]. To convert the comparatively innocuous substance 5-fluorocytosine (5-FC) into the cytotoxic compound 5-FU in situ, for instance, strains of *Bifidobacterium* have been modified to express cytosine deaminase [244]. In mice, co-administration of 5-FC with a *B. infantis* strain that produces cytosine deaminase-e dramatically suppressed tumor growth [245]. Similarly, to inhibit tumor growth in a mouse model, Li et al. built an EcN model to create cytotoxic chemicals, such as glidobactin, luminmide, and colibactin [246]. Targeting specific cancer cells has been demonstrated by several bacterial toxins. Targeted gene therapy against colon cancer cell lines overexpressing claudin-3 and/or − 4 can be achieved by using a translationally optimized *Clostridium perfringens* enterotoxin expression vector (optCPE), which enables the quick and efficient removal of tumor cells in both in vivo and in vitro [247].

### Phage therapy

Phage therapy is an excellent strategy to treat infections. It has received notable attention for the effective eradication of multidrug-resistance bacteria and treating diseases [248]. By modularly switching phage tail components, a generalizable and effective technique for phage genome engineering was developed to reroute *E*. *coli* phage scaffolds to target pathogenic *Yersinia* and *Klebsiella* bacteria, and vice versa. The new target microorganisms were effectively eliminated by the synthetic phages [249]. According to a recent study, the lytic five-phage combination reduces inflammation and the severity of the disease by targeting susceptible and resistant members of the *K*. *pneumoniae* (Kp) clade, which is linked to IBD [248, 250]. Multidrug-resistant uropathogenic *E. coli* (UPEC) represents a significant global health system danger [251, 252]. According to the results of the phage-host interaction, the phage vB_Ec_ZCEC14 of the Straboviridae family has potent and consistent antibacterial activity against antibiotic-resistant UPEC at lower doses (MOI 0.1) [253]. In a 28-day double-blind, placebo-controlled crossover experiment, people ranging in weight from normal to overweight took bacteriophages. Phage eating was shown to be associated with decreases in fecal *E. coli* levels. Additionally, a lower percentage of taxa closest related to *Clostridium perfringens* and an increase in members of the butyrate-producing genus *Eubacterium* were noted [254]. For a duration of 28 days, healthy persons who self-reported experiencing GI distress were included in another study. They were instructed to take one 15-mg capsule containing four different strains of bacteriophages (LH01-Myoviridae, LL5-Siphoviridae, T4D-Myoviridae, and LL12-Myoviridae) or a placebo. While receiving the therapy and the placebo, participants also reported notable improvements in a number of GI discomfort symptoms [255]. *Fusobacterium nucleatum* (Fn) preferentially generates immunosuppressive myeloid-derived suppressor cells (MDSCs) to impede the host's anticancer immune response. This process is how Fn contributes to CRC. Using silver nanoparticles (AgNP) together with an M13 phage that selectively binds Fn demonstrated exceptional efficiency against Fn. According to research conducted in vivo and in vitro, M13@Ag therapy can scavenge Fn in the gut and reduce MDSC proliferation at the tumor site [256]. Using a lytic phage cocktail showed potential for targeting *Klebsiella pneumoniae* and *Enterococcus gallinarum* to treat primary sclerosing cholangitis [257].

## Conclusion and future prospects

The AM emerges as a crucial contributor to human health, challenging conventional perceptions. It serves as a sanctuary for beneficial bacteria, crucial for gut health and immune system modulation. While direct evidence of the AM’s role in different diseases is still developing, the concept of using it as a therapeutic reservoir is promising and warrants further investigation. Research revealed that autoimmunity might be induced by FMT when transferred from autoimmune patients to healthy mice [89]. It is assumed that healthy subjects contain a balanced GM. When a transferred microbe by FTM causes autoimmune disease in healthy subjects, it means the transferred microbes might become dominant over the commensal flora. Issues like this could open a new era of research. Moreover, there are conflicting outcomes in research when working on the role of GM in some disease progression like PD. The conflicting evidence on the relationship between appendectomy and PD underscores the complexity of the disease and the need for a nuanced understanding of the GBA. Therefore, clinicians should consider appendectomy history when assessing PD, but more studies are required to clarify this relationship and explore therapeutic possibilities. Postbiotics could signify an advanced therapeutic approach if the microbial metabolites that are part of them can work synergistically on the target site (like the gut-vascular axis). In this instance, the intravenously supplied metabolites of bacteria might enter the bloodstream and provide direct action on, which is a novel indirect upgrade on vascular function brought about by the restoration of the intestinal microbiota’s homeostasis [211]. However, it is a promising advancement of science and could be applied to investigate other diseases as well.

Future research should focus on several key areas. There is a need for well-designed clinical trials and longitudinal studies to explore the role of AM in health and disease comprehensively. These studies should aim to understand the long-term effects of manipulating AM and its impact on various health conditions, including autoimmune diseases, neurodegenerative disorders, and metabolic syndromes. Research on gut fungi and parasites is significantly lacking compared to the gut bacteria. Future research should aim to elucidate the roles of gut fungi, parasites, and viruses, and their role in disease progression and their potential as therapeutic targets. Understanding the interactions between these microorganisms and the host immune system could lead to novel therapies for various diseases. The targeted change of microbiota by either restoringfunctions that are missing or eliminating functions which are harmful, may result in new methods to avoid or cure various ailments [21]. Future research promises innovative therapies harnessing the appendix's microbiota to enhance immune function and combat disease. Despite current limitations, particularly in human studies, understanding the appendix's complex symbiosis with the GM holds transformative potential for personalized medicine. As we unravel its complexities, the appendix represents the profound interplay between humans and their microbial counterparts, offering avenues for revolutionary healthcare advancements.

## Data Availability

No datasets were generated or analysed during the current study.

## Abbreviations

AM: Appendix microbiome
AD: Auto-immune disease
ALS: Amyotrophic lateral sclerosis
ASD: Autism spectrum disorder
CD: Crohn’s disease
CNS: Central nervous system
CRC: Colorectal cancer
CVDs: Cardiovascular diseases
FMT: Fecal microbial transplantation
GBA: Gut-brain axis
GI: Gastrointestinal
GM: Gut microbiota
IBD: Inflammatory bowel disease
LDL: Low-density lipoprotein
LPS: Lipopolysaccharides
NDs: Neurological disorders
PD: Parkinson disease
PKC: Protein kinase C
SCFAs: Short-chain fatty acids
TLR: Toll-like receptor
UC: Ulcerative colitis
